# Supplementary figures and images for: A recyclable and light-triggered nanofibrous membrane against the emerging fungal pathogen Candida auris
Source: PLoS Pathog. 2022 May 25;18(5):e1010534. doi: 10.1371/journal.ppat.1010534 (PMC9173615; doi:10.1371/journal.ppat.1010534)

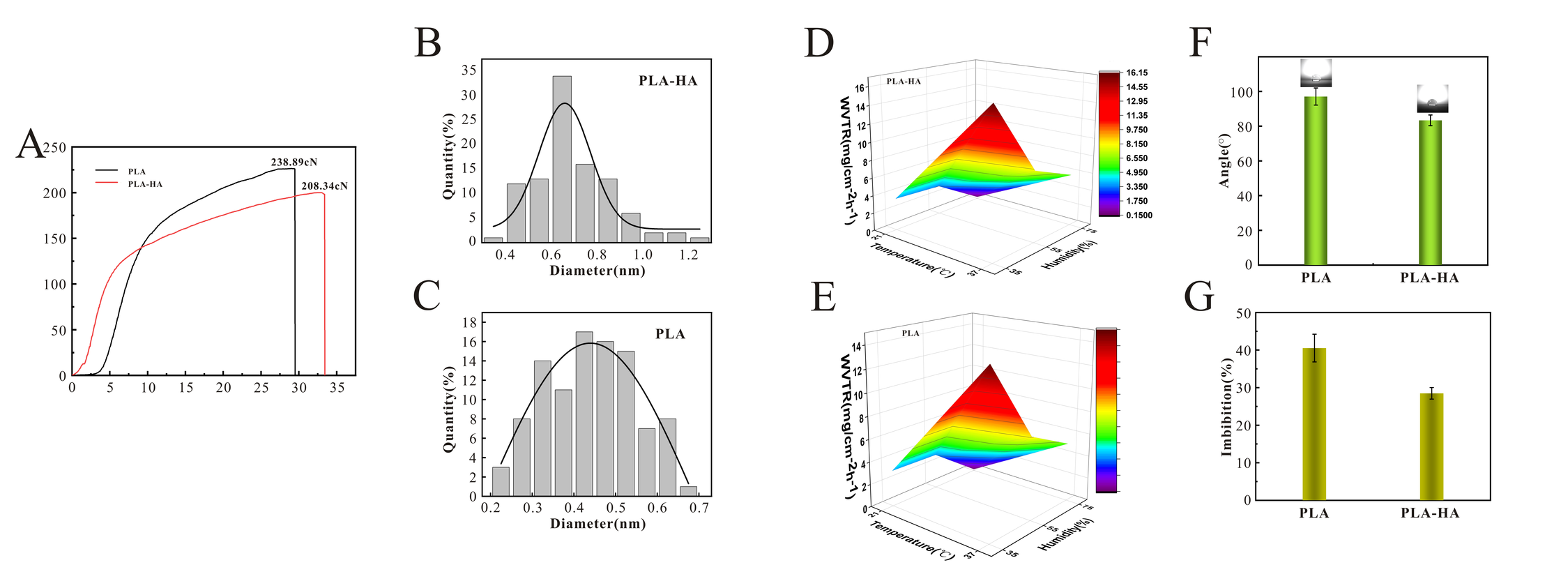

Supplement: S1 Fig — (A) The mechanical properties of PLA and PLA-HA. The relationship between the breaking strength and elongation at break of PLA and PLA-HA was detected. (B&C) The diameter distributions of PLA-HA and PLA. (D&E) WVTR of PLA-HA and PLA with different detected humidities and temperatures. (F) Water contact angle measurements of PLA and PLA-HA. (G) The imbibition rate of PLA and PLA-HA. Data are presented as the mean ± s.d. (TIF) [file ppat.1010534.s001.tif]

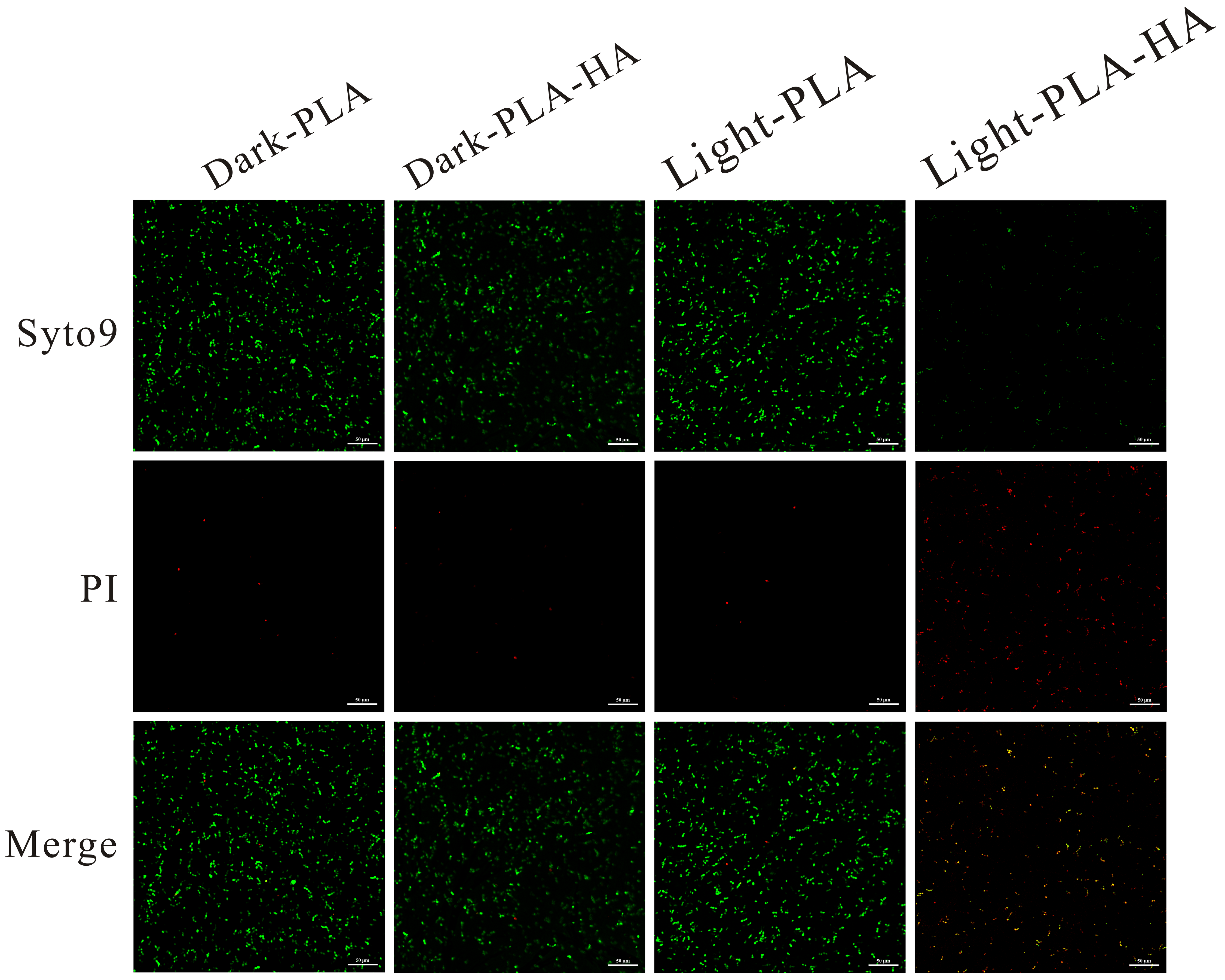

Supplement: S2 Fig — (TIF) [file ppat.1010534.s002.tif]

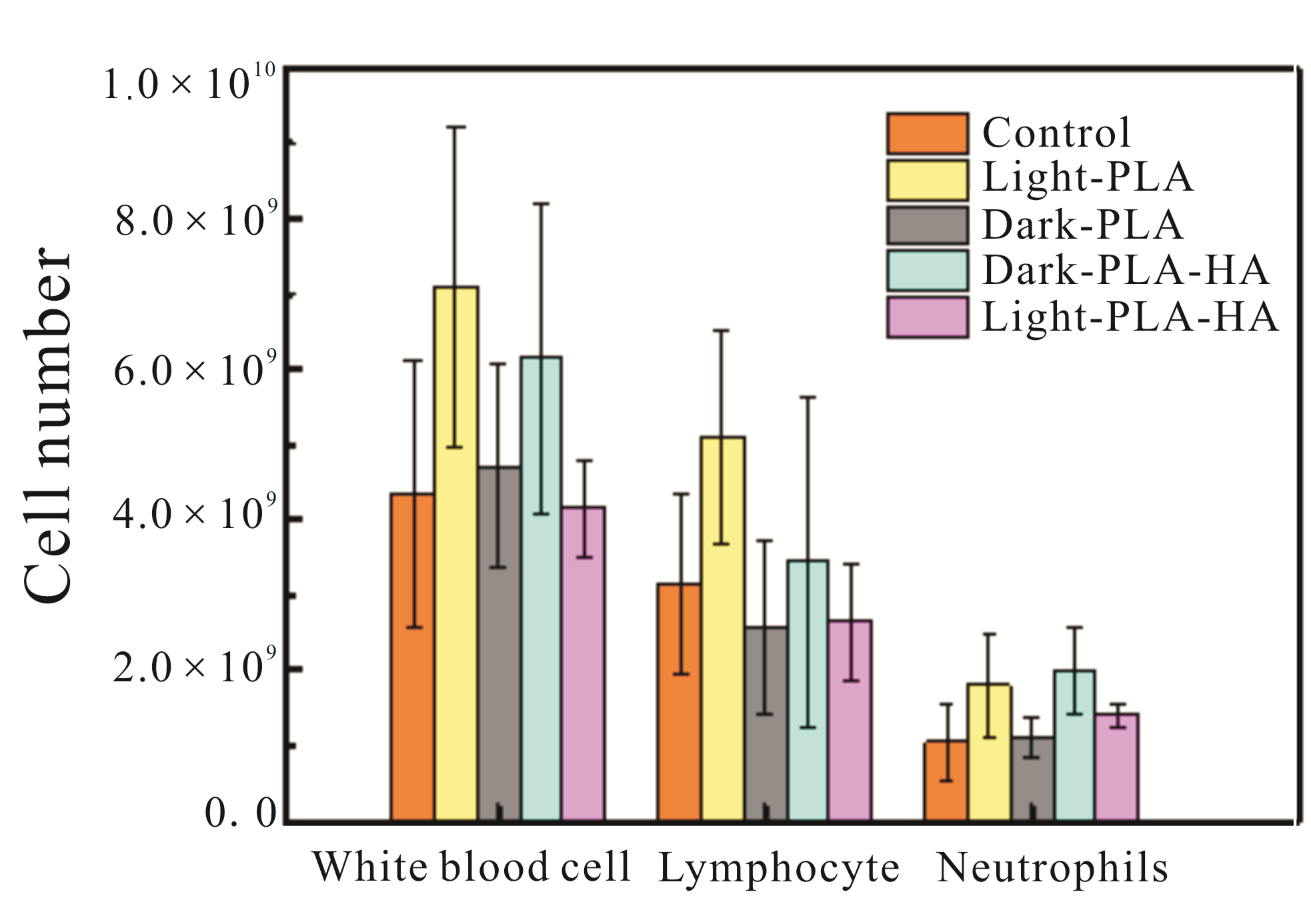

Supplement: S3 Fig — (TIF) [file ppat.1010534.s003.tif]
