# Supplementary material for: A recyclable and light-triggered nanofibrous membrane against the emerging fungal pathogen Candida auris
Source: PLoS Pathog. 2022 May 25;18(5):e1010534. doi: 10.1371/journal.ppat.1010534 (PMC9173615; doi:10.1371/journal.ppat.1010534)
Supplement: S1 Table — (DOCX) [file ppat.1010534.s004.docx]

Table S1 WVTR of PLA-HA and PLA with different detected humidity and temperature

| Temperature | Humidity | PLA | PLA-HA |
| --- | --- | --- | --- |
| 21 ℃ | 35% | 3.18 ± 0.19 | 3.61 ± 0.93 |
|  | 55% | 3.09 ± 0.21 | 3.31 ± 0.39 |
|  | 75% | 0.12 ± 0.05 | 0.19 ± 0.07 |
| 37 ℃ | 35% | 14.06 ± 3.18 | 16.10 ± 3.60 |
|  | 55% | 7.20 ± 3.09 | 7.95 ± 3.32 |
|  | 75% | 5.18 ± 0.12 | 5.87 ± 0.12 |

Data represent the mean ± SD of three replicates.
