# Supplementary material for: A recyclable and light-triggered nanofibrous membrane against the emerging fungal pathogen Candida auris
Source: PLoS Pathog. 2022 May 25;18(5):e1010534. doi: 10.1371/journal.ppat.1010534 (PMC9173615; doi:10.1371/journal.ppat.1010534)
Supplement: S1 Text — (DOC) [file ppat.1010534.s006.doc]

**Electrospinning**

10 wt% PLA was dissolved in the mixture containing DMF and DCM in a ratio of 1:4, After stirring for 12 h at room temperature, 0.2 wt% HA was evenly added into the above mixture and continually stirred for another 2 h at 25°C. The solution was electrospun into nanofibrous membrane through a manual assembly electrospinning system (provided by R&D Lab of Functional Fibers of Sichuan University) with the following parameters: the solution flow rate was 1 mL/h, the range of applied positive and negative voltages was 15 to 20 kV and 0.5-0.6 kV, respectively, and the distance between the needle tip and the target was 10-11 cm. All electrospinning processes were conducted under an ambient condition. The obtained nanofibrous membranes were named as PLA (without HA) and PLA-HA.

**Characterization of Electrospun Nanofiber Membranes**

The morphology of electrospun nanofibers was observed by a scanning electron microscope (SEM, Hitachi SU3500, Japan) after coating with gold. The average diameter was determined based on the SEM images of each sample, and at least 100 different fiber segments were randomly selected and their diameters were measured using *Image J* software. The surface wettability of the nanofibrous membrane surfaces was characterized through a contact angle tester (HarkeSPCAX1, China) using the sessile drop method. The mechanical properties of PLA-HA and PLA were tested by an electronic single yarn strength tester (YM061, China). The thickness between film specimens did not exceed 100%. Additionally, the tensile rate, width and fixture distance were set to 100 mm/min, 3 mm and 20 mm, respectively. Finally, the average of breaking force (cN) and elongation at break (%) were recorded. To measure the imbibition rate and water vapor sorption, squared specimens (1 cm × 1 cm) of each film were cut and obtain the initial mass (W_0_) of each sample was obtained using an electronic balance. Briefly, the films were soaked with PBS for 12 h, drained by filter paper. Then, the wet mass (Wt) was weighed. The imbibition rate was calculated using the following equation: Imbibition rate (%) = (W_t_ − W_0_) /W_0_ × 100%. For the water vapor transmission rate (WVTR), the bottles with ultra-pure water were sealed by nanofibrous films. The moisture consumption through film specimens (M_0_) were recorded. The area of sample (S) and evaporation time (T) was also measured. In this process, humidity and temperature for each test was set for 35%, 55%, 75%, and 21°C, and 37°C, respectively. Finally, the WVTR (g·m^−2^·d^−1^) was calculated according to the following equation: WVTR (%) = M_0_/S/T × 100%. All experiments were independently repeated in triplicate.
