# Supplementary material for: A recyclable and light-triggered nanofibrous membrane against the emerging fungal pathogen Candida auris
Source: PLoS Pathog. 2022 May 25;18(5):e1010534. doi: 10.1371/journal.ppat.1010534 (PMC9173615; doi:10.1371/journal.ppat.1010534)
Supplement: Attachment — Submitted filename: Manuscript for pLos-pathogens.doc [file ppat.1010534.s007.doc]

# A recyclable and light-triggered nanofibrous membrane against the emerging fungal pathogen *Candida auris*

Xinyao Liu^1,3,4^, Chuan Guo^2^, Kaiwen Zhuang^1,3,4^, Wei Chen^5^, Muqiu Zhang^1,3,4^, Yalin Dai^6^, Lin Tan^2*^, Yuping Ran^1,3,4*^

1 Department of Dermatovenereology, West China Hospital, Sichuan University, Chengdu, 610041, China

2 College of Biomass Science and Engineering, Sichuan University, Chengdu, 610065, China

3 Laboratory of Dermatology, Clinical Institute of Inflammation and Immunology (CIII), Frontiers Science Center for Disease-related Molecular Network, West China Hospital, Sichuan University, Chengdu, 610041, China

4 Academician Workstation of Wanqing Liao, West China Hospital, Sichuan University, Chengdu, 610041, China

5 Department of Physics, The University of Texas at Arlington, Arlington, TX 76019-0059, USA

6 Division of Clinical Microbiology, Department of Laboratory Medicine, West China Hospital, Sichuan University, Chengdu, 610041, China

* Corresponding authors:

Yuping Ran, Department of Dermatovenereology, West China Hospital, Sichuan University, Chengdu 610041, China. E-mail address: ranyuping@vip.sina.com. ORCID: 0000-0002-9382-0571

Lin Tan, College of Biomass Science and Engineering, Sichuan University, Chengdu, 610065, China. E-mail address: tanlinou@scu.edu.cn. ORCID: 0000-0001-8440-5643

**Key words:** Antimicrobial photodynamic therapy; *Candida auris*; Hypocrellin; Nanofibrous membrane

# Abstract

The emerging "super fungus" *Candida auris* has become an important threat to human healthy due to its pandrug-resistance and high lethality. Therefore, the development of novel antimicrobial strategies is essential. Antimicrobial photodynamic therapy (aPDT) has excellent performance in clinical applications. However, the relevant study on antifungal activity and the mechanism involved against *C. auris* remains scarce. Herein, a recyclable and biodegradable polylactic acid-hypocrellin A (PLA-HA) nanofibrous membrane is newly developed. *In vitro* PLA-HA-aPDT could significantly reduce the survival rate of *C. auris* plankton and its biofilms, and the fungicidal effect of the membrane is still significant after four repeated uses. Simultaneously, PLA-HA exhibits good biocompatibility and low hemolysis. *In vivo* experiments show that PLA-HA-aPDT can promote *C. auris*-infected wound healing, reduce inflammatory response, and without obvious toxic side-effects. Further results reveal that PLA-HA-aPDT could increase endogenous reactive oxygen species (ROS) levels, leading to mitochondrial dysfunction, release of cytochrome C, activation of metacaspase, and nuclear fragmentation, thereby triggering apoptosis of *C. auris*. Taken together, the efficacy, safety and antifungal activity make PLA-HA-aPDT a highly promising antifungal candidate for skin or mucous membrane *C. auris* infection.

## Author summary

It is urgent to develop new antifungal strategies to face the problem of *Candida auris* infection and drug resistance. Previous studies have revealed that antimicrobial photodynamic therapy (aPDT) based on natural products, like hypocrellin A (HA), is a promising method in clinical applications. However, equivalent studies of aPDT on antifungal activity and its mechanism against *C. auris* remains scarce. Herein, we successfully prepared a recyclable, biodegradable, and light-driven antifungal PLA-HA nanofibrous membrane through the electrospinning technique. *C. auris*-infection has been treated by aPDT *in vitro* and *in vivo* for the first time, especially HA mediated aPDT. *In vitro* and *in vivo* experiments have provided sufficient lines of evidence that PLA-HA is a promising antifungal material for superficial *C. auris* infections due to its antifungal effect and excellent biocompatibility. Notably, there still remains a very high antifungal activity after utilizing PLA-HA four times. In addition, this study clarifies that the anti-*C. auris* mechanism of PLA-HA, namely the PLA-HA mediated aPDT, is attributed to the formation of intracellular ROS, resulting in a dysfunction of mitochondria and the decline of the mitochondrial transmembrane potential, releasing of cytochrome C from mitochondria to cytoplasm, promoting the activation of metacaspase, inducing nuclear condensation and the fragmentation of *C. auris*, thus triggering yeast cell apoptosis. This study would lay a foundation for developing new antimicrobial nanofibrous dressings mediated by aPDT, and provide an alternative strategy for the treatment of local fungal infectious diseases.

# Introduction

With the widespread application of broad-spectrum antimicrobial agents and immunosuppression, fungal infection has become a major global health threat [1]. The related challenge of the emergence of fungicide resistance is also an increasingly serious problem worldwide [2]. Comparing with various antibiotics for bacterial treatment, azoles, polyenes and echinocandins are the only three kinds of antifungal drugs clinical available. The long-term use of antifungal drugs can easily cause the emergence of newly and multiple-resistant strains. *Candida auris*, first isolated from the ear discharge of a female Japanese patient in 2009, is a newly emerged member of the Candida family [3]. As a new opportunistic pathogen, *C. auris* can cause both superficial and life-threatening infections in immunocompromised hosts, especially nosocomial infections. In the past decade, infections caused by *C. auris* have become a global threat due to its rapid emergence worldwide and multidrug resistance properties [4]. Based on the above reasons, *C. auris* is also called “Super fungus”, which has been reported that the proportion of fluconazole-resistance strains even exceeds 90% [5]. Therefore, it is imperative to identify alternative antifungal agents or strategies that are effective against *C. auris* infections.

Antimicrobial photodynamic therapy (aPDT) involves non-toxic photosensitizers (PS) and suitable light source to induce the production of reactive oxygen species (ROS) which can kill the microbial pathogens [6,7]. Whether *in vitro* or *in vivo*, numerous studies have demonstrated that ROS can inactivate many microorganisms regardless of the drug-resistance [8-10]. To date, many studies report that aPDT can overcome the resistance in fungi and bacteria, even if microbial pathogens can protect against the damaging effects of ROS and overcome toxicity of photooxidative stress generated by aPDT in some degree [11-13]. In addition, the type of PS is the decisive factor of aPDT efficiency [14,15].

Hypocrellin A (HA), which is generally regarded as a new type of PS, belongs to the class of perylenequinonoid compounds [16]. This lipid-soluble pigment is one of the main secondary metabolites (SMs) produced by the Chinese medicinal fungi *Shiraia bambusicola* P. Henn. and *Hypocrella bambusae* (Berk. & Broome) Sacc., the fruit bodies of which can unblock meridians, dissolve phlegm and arrest cough [16,17]. It is well known that HA plays an important role in PDT for anticancer [18], antiviral [19] and antimicrobial [20-22] treatments, thanks to its production of singlet oxygen and semi-quinone free radicals under light irradiation. However, studies on the antifungal photodynamic activity of HA are limited, only focused on *Candida albicans* [21,23]. As a newly discovered “Super fungus”, aPDT or HA treatment on *C. auris* infection has never been reported previously.

Acute and chronic skin wounds are important infective routes for *C. auris* to invade human localized tissue or cause systemic infection [4]. Therefore, anti-infective wound dressings are beneficial to heal wounds and promote tissue repair [24,25]. In antifungal-drug delivery materials, electrospun nano-membranes offer greater air permeability, higher porosity and surface area, higher loading capabilities of PS, making them an excellent choice for withstanding the invasion of exogenous microorganisms [26,27]. Severyukhina *et al.* used chitosan and the second-generation PS to prepare an electrospun nanofiber membrane, and achieved local PDT sterilization with visible light irradiation [28]. In addition, Si *et al.* reported a green bio-based nanofibrous membrane with antimicrobial activity, which could be repeatedly sterilized by sunlight excitation [29].

In this study, we have prepared a novel type of polylactic acid-hypocrellin A (PLA-HA) nanofibrous membrane and tested the PLA-HA based aPDT effects on *C. auris* treatment. Firstly, fungal culture, biofilm construction, morphological observation, and animal models of skin wound infection were used to clarify the role of PLA-HA nanofibrous membrane-aPDT on *C. auris*. Secondly, the ROS levels and apoptosis hallmarks phosphatidylserine ectropion, mitochondrial transmembrane potential, DNA fragmentation, nuclear condensation, metacaspase and cytochrome C activity in *C. auris* were examined to investigate the cellular mechanism of PLA-HA-aPDT killing (Fig 1). This is the first application of nanofibrous membrane with non-toxic photosensitizer for photodynamic therapy against *C. auris.*

# Fig 1. A Schematic illustration for the synthetic route of PLA-HA membrane and its antifungal applications in *C. auris* planktonic cells and biofilms, as well as the corresponding infected wound healing.

# Results and Discussion

# Preparation and Characterization of Electrospun Nanofibrous Membrane

In order to enhance the stability and controllability of HA, we prepared PLA-HA nanofibrous membrane (Fig 2). As shown in Fig 2, the color of PLA-HA changed from white (the color of PLA) to red (the color of HA) due to the adding of HA [30]. Micrographs of PLA and PLA-HA nanofibrous membranes were observed by scanning electron microscopy (SEM) (Fig 2), in which the corresponding average fiber diameters were 423 and 699 nm, respectively (S1B Fig, S1C Fig). The surface morphology of fibers presented randomly three-dimensional, uniform and beads-free (Fig 2), which was in accordance with the criteria of an excellent electrospun nanofibrous membrane [31]. After adding HA, the blended solution turned thicker and interactions between molecular chains and solvents turned larger, which led to the increase of the average diameter of PLA-HA. Other than these changes, we observed no pronounced differences in structure or morphology of the fibers.

**Fig 2.** Visual appearance and surface morphologies of PLA and PLA-HA nanofibrous membranes.

Appropriate mechanical properties should be an important index for ideal wound dressing [32]. Fig S1A exhibits the mechanical properties (breaking force and elongation at break) of the obtained nanofibrous membranes. It was reported that the elongation of human skin varies in range of 17-207% for elongation at break [33]. In this study, the elongation at break of each membrane was 28.18% for PLA and 33.8% for PLA-HA. Therefore, the mechanical properties of our membranes are in acceptable elongation range for wound dressing. Taken these together, the increase of the average diameter of PLA-HA led to the decrease of adhesion degree among fibers and the increase of elongation at break, which consisted with the opinion of the bonding between the components of the membranes exhibiting a significant effect on the mechanical properties [32].

Water vapor transmission rate (WVTR) reflects the capacity of moisture management in wound area of membrane. It is known that high values of WVTR can accelerate the drying process, while a low WVTR would result in the exudates accumulating and microbial infections, hindering the wound healing process [34]. The range of WVTR for human skin is 0.024-0.192 g·cm^-2^·24 h^-1^, and WVTR of exposed wound is 0.480 g·cm^-2^·24 h^-1^ [35]. The WVTR of our membranes in this study was in the intermediate. Our results showed increasing of humidity leading to decreasing of WVTR, but the opposite happened when increasing temperature. In addition, WVTR of PLA-HA is higher than PLA membrane at the same conditions (S1D Fig, S1E Fig, S1 Table), which indicates that PLA-HA is more beneficial for promoting volatilization of exudates.

An excellent wound dressing should have an ability of absorbing excess exudate from the wound area and maintaining a humid environment for wound healing [34]. Fig S1G shows the imbibition rates of PLA (40.5%) and PLA-HA (28.4%) membrane. As revealed in the Fig S1G, addition of HA to the composite decreased the absorption efficiency. Compared with PLA membrane, PLA-HA membrane is more advantageous to reduce the accumulation of exudate on wound.

In addition, the surface wettability of PLA and PLA-HA membrane were measured by a water contact angle (WCA) system (S1F Fig). The WCA value of the PLA membrane was 97.06°, while it decreased to 83.36° after coating with HA, slightly promoting the hydrophilic property of the membrane specimen.

# *In Vitro* Antifungal Activity

To observe the antifungal activity of PLA-HA, *C. auris* (BJCA001, the first isolated strain in China) was selected [36]. BJCA001 strain was treated with PLA and PLA-HA with or without light irradiation. Agar plate images and quantitative histograms of the colonies are shown in Fig 3A and 3C. Our results show that the PLA and PLA-HA treatments without illumination did not cause any significant toxicity in *C. auris*. However, when illumination was employed for 30 min, the survival rate of the PLA-HA treated strain decreased dramatically with the survival rate lower than 0.1%. Notably, the antifungal activity of PLA-HA is still very effective after reusing for 4 times. Compared with the recent reported antimicrobial materials on HA and its derivatives which fixed on powdery nanocarrier [18,37]. our material exhibits excellent repeatable antifungal activity.

Moreover, the Live/Dead double staining fluorescent dye Syto9/PI was used to further evaluate antifungal activity of PLA-HA (S2 Fig). *C. auris* was stained with Syto9 and PI. The Syto9 with green fluorescence could stain all fungal cells, whereas PI only stained cells with damaged wall. When Syto9 and PI dyes stained a cell at the same time, the fluorescence intensity of Syto9 becomes weaker [38]. As shown in Fig S2, PLA and PLA-HA treated *C. auris* without illumination, PLA treated *C. auris* with illumination were stained with bright green fluorescence, indicating that the fungi had an integrated wall and membrane structure. However, the PLA-HA treated strain exhibited red fluorescence which suggested the cell wall has been damaged.

SEM and TEM were used to observe how PLA-HA kill *C. auris* (Fig 3B). SEM of *C. auris* shows that the surfaces of the healthy blastospores are smooth with elliptic in shape, and the boundaries between blastospores are clearly visible. However, PLA-HA with illumination could destroy the cell wall and membrane of the fungal, resulting in a shrinkage of cells, a conglutination occurred in the adjacent spores. Also, fragments of blastospores are clearly visible in SEM images. From the TEM photographs, the healthy blastospores of *C. auris* in three control groups show regular shapes, with a clear outlined cell walls and cytoplasmic membranes. In the cytoplasm, organelles such as nucleus and lipid inclusions were regularly distributed. Meanwhile, the shape of cells of PLA-HA treated *C. auris* is irregular and severely damaged as seen by TEM, the permeability of cell was significantly increased, contents of destroyed fungal flowed out of the cell. Furthermore, some organelles disappeared, some cytoplasms were disintegrated into empty bubbles, the nucleus was fragmented, and some cell walls and membranes were broken and discontinuous.

In addition, the cytocompatibility of the wound dressings plays a pivotal role on wound healing. We used mouse fibroblast (L929) cells to examine the toxicity of PLA and PLA-HA without illumination. The results of CCK-8 assay indicated that both PLA and PLA-HA did not cause the obviously cytotoxicity toward L929 cells after 24, 48 and 72 h of incubation (Fig 3E).

Hemolysis ratio (HR) is an important factor for the hemo-compatibility of biomaterials. There is no obvious hemolysis of wound dressing when the dressing directly contacted with blood *in vivo* [39]. Therefore, hemolysis experiment was used to investigate the effect of PLA and PLA-HA on the rupture and lysis of red blood cells (RBCs). As shown in Fig 3D, HR of PLA is 1.51% and the other is 0.89%, indicating that both PLA and PLA-HA did not cause obvious hemolysis and have an excellent blood biocompatibility.

**Fig 3.** *In vitro* antifungal activities and biosafety of PLA-HA and PLA. A. Spread plate results of *C. auris* growth under different conditions. The images from left to right represented PLA and PLA-HA were repeat used for four times. B. SEM and TEM images of *C. auris* under different conditions. CM indicated cytoplasmic membrane, CW represented cell wall, N meant nucleus and L referred to lipid inclusion. C. Corresponding survival rate results of *C. auris* with PLA and PLA-HA treatment (n = 3). D. Hemolysis assay. The inset showed the image directly observed after adding PLA and PLA-HA for 2 h. Distilled water was used as a positive control, and PBS was used as a negative control. E. Cell toxicity evaluation of PLA and PLA-HA on mouse fibroblast L929 for 24 h, 48 h and 72 h. Data are presented as the mean ± s.d.

# Capacity of *C. auris* Biofilm Eradication

Biofilm formation plays an important role in virulence and pathogenicity of *C. albicans,* which can result in death [40]. Also, biofilm formation is often correlated with increased drug resistance among *Candida* species [41]. Fig 4A and 4B show the growth curves of *C. auris* biofilm according to fluorescence stain assay and XTT reduce method. From Fig 4A, we can see that this strain formed by clumped cells without filamentation. As time goes on, the gaps between cell clusters of biofilms have become narrower within 24 h, marked the mature of biofilm. This result was consistent with metabolic activity of biofilm (Fig 4B), which increased rapidly within 24 h but slowly later on.

Therefore, the 24 h biofilm of *C. auris* was selected to assess the biofilm eradication activity of PLA and PLA-HA. As shown in Fig 4C, the inhibition rate of PLA-HA treated biofilm of *C. auris* with illumination is up to 77%, as compared to the other control groups.

Subsequently, the Live/Dead double staining fluorescent dye Syto9/PI was used to further evaluate the biofilm eradication activity of PLA and PLA-HA; the dyeing principle is mentioned above. Compared to other groups, the PLA-HA treated biofilm of *C. auris* with illumination presented red fluorescence, indicating the biofilm was badly damaged by CLSM observation (Fig 4D). Fig 4E shows the result of biofilm eradication activity of PLA and PLA-HA by SEM observation. Compared with other groups, the biofilm turned sparse and the strains were shrunken after treatment of PLA-HA under 470 nm laser. Taken together, PLA-HA aPDT could eradicate biofilm of *C. auris*, efficiently.

**Fig 4.** Biofilm formation and capacity of *C. auris* biofilm eradication. A. Observed of *C. auris* biofilm formation under different time using Styo9 staining. Scale = 200 μm. B. Growth curve of *C. auris* biofilm by XTT reduce method. C. Capacity of *C. auris* biofilm eradication of PLA and PLA-HA with and without illumination using XTT reduce method. D. Live/Dead staining of *C. auris* biofilm observed by CLSM. Scale = 10 μm. E. SEM images of *C. auris* biofilm with PLA and PLA-HA treatment. Data are presented as the mean ± s.d. n = 3.

***In Vivo* Antifungal Assay**

Due to the excellent antifungal ability *in vitro* with no obvious toxicity of PLA-HA to mouse fibroblast cells, we further evaluated the antifungal ability of samples through photodynamic therapy *in vivo* on *C. auris*-infected mice. The infectious and therapeutic processes are shown in Fig 5A. The results show that the PLA-HA mediated aPDT remarkably promoted wound healing as evidenced by the reduced wound infection size, with the wound healing rate increasing about 10% when compared to that of other groups (Fig 5C, Fig 5D). After treatment, the fungi in the infected wound sites of different groups were quantified using the agar plate method. The number of *C. auris* colonies in PLA-HA treated mice was less than 2%, as shown in Fig 5B and 5D, while the fungi have grown well in other groups. This result suggested that the PLA-HA mediated aPDT could decrease *C. auris* burden in the infected mice skin wounds. As shown in Fig S3, there were no significant differences of white blood cells (WBCs), lymphocytes (LYs), and neutrophils (NEs) between the PLA-HA mediated aPDT group and the other groups. Also, the above indexes were in the normal range. This result demonstrated that *C. auris* in our study only caused superficial infection, not involved system infection.

To further determine the anti-infective efficacy of the PLA-HA mediated aPDT, histopathology changes in *C. auris*-infected tissue sites at the end of treatment were examined. H&E staining on skins was used to investigate the wound healing process (Fig 5E). The epidermis and dermis structure in infected tissue in different groups were damaged at various levels. In the PLA-HA mediated aPDT group, an intact epidermal layer and some new hair follicles were formed, and a small number of inflammatory cells appeared. In contrast, in other groups numerous inflammatory cells, local infected tissue necrosis, and bleeding were clearly visible. PAS staining can help to diagnose fungal infection in biopsies and has been considered the most sensitive method for diagnosis [42]. In our study, PAS staining was utilized to observe *C. auris* in tissue sites. The PLA-HA mediated aPDT group presented negative results of PAS, but in other groups the trough-like, circular yeast cells were visible, and the color of cell wall presented burgundy which indicated PAS positive (Fig 5E). Furthermore, the expression level of the inflammatory cytokine interleukin-6 (IL-6) in the wound sites was detected by immunohistochemistry (Fig 5E, Table 1). IL-6 positive staining appeared pale brown, the corresponding average optical density (AOD) could reflect the positive expression level of IL-6, indirectly. In PLA-HA mediated aPDT group, the expression level of IL-6 was much lower than the other groups, indicating that the inflammatory response in mice was significantly controlled by PLA-HA under illumination. All the above experiments proved the strong antifungal activity of PLA-HA mediated aPDT *in vivo*. To further investigate the biocompatibility of PLA and PLA-HA under illumination *in vivo*, the heart, liver, spleen, lung, and kidney of mice were stained by H&E after therapy, and no obvious pathological abnormalities were observed (Fig 5F). This result proved that the PLA-HA mediated aPDT showed no obvious side-effect to mice *in vivo*.

**Fig 5.** Antifungal efficacy and compatibility of PLA-HA in treating cutaneous *C. auris* infections. A. Schematic diagram of cutaneous wound infection with *C. auris* and treatment with PLA-HA. Mice were evaluated on the 3rd and 21st day. B. Survival rate of *C. auris* in the infected wound sites treated by PLA-HA and PLA. C. Corresponding wound healing rate of mice in different groups. D. Representative photographs of wounds and fungal burden of PLA-HA and PLA treated mice in day 3 and day 21. E. Infected skin wound tissue evaluated by H&E staining (day 21, scale = 100 μm), PAS staining (day 3, scale = 50 μm) and IHC of IL-6 (day 3, scale = 20 μm) after PLA-HA treatment. F. H&E staining of lung, liver, spleen, kidney and heart in different groups after treatment (scale = 20 μm). Data are presented as the mean ± s.d

Table 1 The expression level of IL-6 in *C. auris*-infected tissue sites of different groups

Data represent the mean ± SD of five replicates.

| Groups | Average optical density (AOD) |
| --- | --- |
| Control | 0.019783166 ± 0.0017 |
| Dark-PLA | 0.012174187 ± 0.0035 |
| Dark-PLA-HA | 0.012669057 ± 0.0029 |
| Light-PLA | 0.014977776 ± 0.0026 |
| Light-PLA-HA | 0.001477313 ± 0.0042 |

# Study of Antifungal Mechanism

The production of ROS may cause a direct damage to yeast cells since they induce and regulate apoptosis [43]. Therefore, the capacity of generating extracellular and intracellular ROS of PLA-HA directly determine the efficacy of PDT. In this study, the fluorescent probe DCFH-DA, an indicator of ROS, was used to detect intracellular ROS levels (Fig 6A), and the chemical probe of DPBF was utilized to assess the extracellular ROS levels. As shown in Fig 6B, PLA-HA aPDT group emitted a strong green fluorescence, while other groups showed no fluorescence. Fig 6C demonstrated the fluorescence intensity of DCF in PLA-HA mediated aPDT group increased at least 80.95% when compared with other groups. This result suggests that the PLA-HA mediated aPDT produced ROS accumulation in *C. auris*. Fig 6D demonstrated that the absorbance of DPBF decreased rapidly in the PLA-HA mediated aPDT group. The decrease rate of DPBF was proportional to the increase of irradiation time within 30 min, which reached 23% in maximum. This result confirms that the PLA-HA mediated aPDT could efficiently generate extracellular ROS. The extracellular and intracellular results revealed that PLA-HA mediated aPDT possessed a strong ability of generating ROS. On the other hand, iodide has highly specific reaction with ^1^O_2_ to form I^3-^ [15], and the oxidation degree was directly proportional to the concentration of ^1^O_2_ in an oxygen saturated solution [44]. As shown in Fig 6E, the color of solution changed from white to pale yellow due to intensify of oxidation. When PLA-HA was exposure to 470 nm laser, two new absorption peaks of 287 and 352 nm appeared and gradually increased linearly in an illumination time-dependent manner because of the increased I^3-^ [44]. This result also demonstrated that the PLA-HA mediated aPDT could generate singlet oxygen.

**Fig 6.** ROS level of PLA-HA mediated aPDT. A. The detected principle of intracellular ROS. B. DCFH-DA detected by fluorescence microscope. C. Change of intracellular ROS level treatment by PLA-HA. D. Absorbance of DPBF treated with PLA-HA. E. UV-visible spectroscopic monitoring of the photooxidation of KI to I^3-^ by PLA-HA. Data are presented as the mean ± s.d.

Mitochondria are an important target of ROS. Usually, an abnormal increase in ROS level would induce the mitochondrial damage and alter the mitochondria membrane potential, which can be regarded as the first event of apoptosis [45]. In general, high MMP JC-1 accumulates in the mitochondrial matrix to form JC-1 aggregates, emitting red fluorescence. When MMP decrease, JC-1 cannot gather in the mitochondrial matrix, and in the form of JC-1 monomers, which generates a green fluorescence [37]. In our study, the ratio of JC-1 aggregates/monomers was lower in the PLA-HA mediated aPDT treated *C. auris* cells than control groups (Fig 7C), indicating a loss of MMP and the mitochondrial damage.

Next, Annexin V/PI staining and FACS analysis were employed to verify the pathway of cell death induced by the PLA-HA mediated aPDT. Phosphatidylserine (PS) often exists on the inside of the plasma membrane, while in the apoptotic and necrotic cells, PS is exposed on the outside. Thus, this index is regarded as an early marker of apoptosis in fungi [46]. Annexin V can specifically bind to externalized PS, and PI only stained damaged cells [45]. As shown in Fig 7B, the control groups show a large viable cell population with a few staining for apoptotic cells (about 30% apoptotic cells). Whereas the PLA-HA mediated aPDT treated *C. auris* cells present a large number of apoptotic cell populations (71.94% apoptotic cells), revealing that the PLA-HA mediated aPDT induced *C. auris* apoptosis.

During the early stages of apoptosis, cytochrome C releases from mitochondria to cytoplasm [45]. Thus, the contents of cytochrome C in mitochondria and cytoplasm were examined in this study. Fig 7D and 7E represent that in the PLA-HA mediated aPDT group, the level of cytosolic cytochrome C distinctly increased while the mitochondrial cytochrome C contents significantly decreased when compared to the control cells. This result suggests that the PLA-HA mediated aPDT triggered the release of cytochrome C from mitochondria to cytoplasm.

Furthermore, the release of cytochrome C to cytosol can activate metacaspases, which are caspase-like cysteine proteases in yeast [21]. The activation of metacaspases is a vital part of inducing early stages of apoptosis, and can be detected by FITC-VAD-FMK staining [45]. If the metacaspases are activated, cells present a green fluorescence. Otherwise, cells appear unstained. Fig 7A shows that the green fluorescence intensity enhanced when *C. auris* were treated by the PLA-HA mediated aPDT, suggesting that the PLA-HA mediated aPDT promoted the activation of metacaspase.

Madeo *et al* demonstrated that two pro-apoptotic factors, Aif1 and Nuc1, could result in DNA fragmentation. Also, DNA fragmentation is known as the hallmarks of apoptotic in late phase [47]. DNA fragmentation of cells is often detected by the TUNEL assay, which can identify apoptotic DNA cleavage by labeling fluorescent dUTP at the 3’-OH ends of DNA [48]. Fig 7A shows that the fluorescence intensity was remarkably enhanced in the PLA-HA mediated aPDT group, suggesting that this group caused DNA fragmentation. DAPI staining was used to further observe the morphological changes in the nucleus [45]. Compared with normal nucleus of control groups, the nucleus of PLA-HA treated yeasts showed a cleaved blue fluorescence or more condensation under 470 nm laser (Fig 7A), implying the PLA-HA mediated aPDT induced nuclear condensation and fragmentation of *C. auris*.

**Fig 7.** Antifungal Mechanism of PLA-HA mediated aPDT. A. TUNEL, DAPI and Metacaspase detected by fluorescence microscope. B. Apoptosis of *C. auris* cells by staining with Annexin V-FITC and PI. C. Mitochondrial membrane potential was evaluated using JC-1 staining. D&E. Cytochrome C release from mitochondria to cytoplasm were assayed after treatment. Data are presented as the mean ± s.d.

**Materials and methods**

**Reagents**

All the chemical reagents were directly used without further purification. N, N-dimethyl formamide (DMF), dichloromethane (DCM), PLA, potassium iodide (KI), 1,3- diphenylisobenzofuran (DPBF), menadione were purchased from Sigma-Aldrich. Absolute alcohol, glutaraldehyde and osmium tetroxide were purchased from Beijing Chemical Reagent Company. An ROS assay kit, JC-1, and one step TUNEL apoptosis assay kit were obtained from the Beyotime Institute of Biotechnology, China. Hypocrellin A (HA, 98%), 2,3-bis(2-methoxy-4-nitro-5-sulfophenyl)-5-[(phenylamino)carbonyl]-2H-tetrazolium hydroxide (XTT) and 3-(N-morpholine) propanesulfonic acid (MOPS) were purchased from Shanghai Yuanye Bio-Technology Co., Ltd.

**Strains and Cultures**

*C. auris* strain (BJCA001) used in this study was gifted from professor Guanghua Huang [36]. This strain was routinely grown overnight at 30°C in yeast peptone dextrose (YPD) medium, which consists of 1% yeast extract (Oxoid, Basingstoke, England), 2% peptone (Solarbio, Beijing, China) and 2% dextrose (Solarbio). For the biofilm of *C. auris* construction, *C. auris* was activated on 9-mm diameter Petri dishes using Sabouraud dextrose agar (SDA), containing 25% dextrose, 1% peptone (Solarbio) and 1.5% agar (Solarbio). The formation of biofilms of *C. auris* were on RPMI 1640 medium, which consists of 1.04% RPMI 1640 powder (Gibco, USA), 3.45% MOPS and 2% dextrose.

**PLA-HA Mediated aPDT on *C. auris***

In brief, overnight *C. auris* cultures were diluted to ~10^6^ CFU/mL in YPD medium, and treated with 1 cm × 1 cm nanofibrous membranes (PLA-HA, PLA) for 30 min with or without 470 nm laser (100 mW/cm^2^) irradiation, respectively. Then, the cultures continued to be incubated for 3 h at 30°C in the dark. The experiments were divided into four groups: PLA with illumination, PLA-HA with illumination, PLA without illumination and PLA-HA without illumination.

**Antifungal Activity Assay**

The antifungal activities of nanofibrous membranes were determined by agar plate dilution method. After aPDT as described above, the treated *C. auris* cells were harvested, and approximately 10^4^ CFU/mL cells were spread onto YPD agar plates. Then, plates were incubated for 24 h at 30°C in the dark after spreading. In order to examine the reutilization of PLA-HA, the nanofibrous membranes were repeatedly used four times. The survival rate was expressed as CFU of the sample group and negative control group. This experiment was independently repeated in triplicate.

**Live/Dead Fungal Fluorescent Imaging**

The phototoxicity of nanofibrous membranes to *C. auris* was evaluated by visualized fluorescent imaging. The viability of fungal cells was determined by Syto9 and propidium iodide (PI) dye (Invitrogen Detection Technologies, USA). Syto9 can stain both dead and live fungi with green fluorescence, while PI with red fluorescence only stains dead fungi. Briefly, after aPDT as described above, fungal cells were washed with PBS and incubated with Syto9 and PI dye (Syto9:PI:PBS = 1.5 µL:1.5 µL:997 µL) in the dark for 30 min at 37°C. After staining, *C. auris* cells were washed with PBS three times, and 10 µL of cells in PBS were dropped onto a glass slide. Finally, stained cells were imaged using confocal laser scanning microscopy (CLSM, LSM880, Zeiss, Germany).

**Investigating Cell Morphologies**

Briefly, after treatment by nanofibrous membranes, suspensions of *C. auris* were centrifuged at 4000 rpm for 5 min and rinsed with PBS three times. Next, *C. auris* cells were fixed with 2.5% glutaraldehyde at room temperature for 4 h. After being washed with PBS, fungal cells were separately dehydrated by sequential treatment with 20%, 50%, 70%, 80%, 90% and 100% ethanol gradients for 10 min. After critical-point drying they were gilded in a vacuum chamber and observed using scanning electron microscopy (SEM, FEI Inspect F50, equipped with a FEG gun operated at 30 kV at high vacuum).

Transmission electronic microscopy (TEM) was used to observe the inner structure of *C. auris.* The treated *C. auris* cells were fixed with a 3% glutaraldehyde overnight at 4°C in advance, and then fixed in 1% osmium tetroxide for 2 h at room temperature. Next, fungal cells were dehydrated in series of graded ethanol solutions and propylene oxide, then infiltrated with resin overnight. Subsequently, the semithin sections were stained with methylene blue. After cut with diamond knife, ultrathin sections were stained with uranyl acetate and lead citrate. Finally, sections were examined with JEM-1400-FLASH Transmission Electron Microscope.

***In Vitro* Cytotoxicity Assay**

Cell toxicity of nanofibrous membranes were evaluated by CCK-8 assay. Nanofibrous membranes (PLA-HA, PLA) were cut into 1 cm × 1 cm size and put into 96-well plates (n = 5), washed with PBS three times. Then, L929 cells were seeded into the above 96-well plates at a density of 1.5 × 10^3^ cells per well, respectively. Besides, the wells without nanofibrous membranes served as the negative control. Next, the 96-well plates were placed in the incubator at 37°C and 5% CO_2_ for 24, 48 and 72 h. After incubation, supernatants were removed and 200 μL CCK-8 solution in serum-free medium were added into the cells. After incubation for another 3 h at 37°C, the absorbance was measured at a wavelength of 450 nm using a microplate reader (SpectraMax M2). The cell viability was calculated using the following equation: Cell viability (%) = (A_S_ − A_B_) /(A_N_ − A_B_) × 100%. Where A_S_, A_N_, and A_B_ referred to the record absorbance of the sample, negative control, and blank control at a wavelength of 450 nm, respectively.

**Hemolysis Assay**

The whole blood was collected from rabbit (Dashuo, China) and then added in PBS buffer (pH = 7.4). After centrifuged at 3,500 rpm for 10 min and rinsed with PBS five times, the red blood cells (RBC) were obtained and dispersed in PBS buffer (10% v/v). Then 1 cm × 1 cm nanofibrous membranes (PLA-HA, PLA) were added into the solution and incubated at 37°C. After 2 h, mixtures were centrifuged to examine the hemolysis. The RBC in PBS and distilled water without adding nanofibrous membranes were used as negative and positive control (n = 5), respectively. The absorbance was measured at a wavelength of 540 nm using a microplate reader (SpectraMax M2). The hemolysis rate (%) = (OD_sample_ − OD_negative control_) /(OD_positive control_ − OD_negative control_) × 100%.

**Biofilm Formation**

Strains were cultured on SDA plates and incubated at 30°C for 24 h. A loopful of cultured yeasts was inoculated in 10 mL of YPD broth, all liquid cultures were kept at 30°C overnight with shaking (200 rpm). Then, 100 µL seed broth was added to 10 mL fresh YPD broth, and continually incubated to log phase at 30°C with shaking (200 rpm). Next, cultures were centrifuged at 4000 rpm for 5 min and washed with PBS three times. Then, the washed cells were re-suspended in RPMI 1640 broth medium and the suspension was adjusted to 1 × 10^6^ cells/mL by blood counting chamber. A total of 200 µL of the suspension was inoculated in flat-bottomed 96-well plates for 2 h at 37°C. After biofilm preliminary formation, the planktonic cells were discarded, washed by PBS three times. Then, 200 µL RPMI 1640 broth medium were added into 96-well plates and the plates containing preformed biofilms continually incubated for 24 h at 37°C. Finally, the planktonic cells were discarded, washed by PBS three times. This experiment was independently repeated in triplicate.

**XTT Reduction Assay**

The XTT reduction method is used for quantification of the microbial respiratory activity and evaluate the toxicity of nanofibrous membranes. Briefly, 1 cm × 1 cm nanofibrous membranes (PLA-HA, PLA) was covered on the biofilms in each well, and were exposed to the 470 nm laser (100 mW/cm^2^) for 30 min. The groups which kept out of the light served as the negative control. Then, 200 µL of XTT/menadione solution (1 mg/mL XTT:0.4 mM menadione:PBS = 40:2:158) was added to each above 96-well plates containing completely dry biofilms (n = 5). The plates were covered with aluminum foil and incubated at 37°C for 2 h. After incubation, the absorbance was measured at a wavelength of 492 nm using a microplate reader (SpectraMax M2). Blank samples were prepared by filling 1 well with 200 µL of XTT/menadione solution.

**Observation of Biofilm**

SEM was used to observe the structure of biofilm, and CLSM was used to observe the Live/Dead biofilm staining by Syto9 and PI dye. Briefly, 50-mm discs were placed on the bottom of 6-well plates. *C. auris* biofilms were formed by adding 2 mL of yeast suspension in RPMI 1640 broth medium (1 × 10^6^ cells/ml) to each well containing discs for 2 h at 37°C. After discarding the planktonic cells, washing the floating cells with PBS three times, 2 mL fresh RPMI 1640 broth medium were added into the above 6-well plates and continually incubated for 24 h at 37°C. After incubation, discs were removed to new 6-well plates and gently washed 3 times with PBS. Subsequently, 1 cm × 1 cm nanofibrous membranes (PLA-HA, PLA) was covered on the disc each well, and were exposed to the 470 nm laser (100 mW/cm^2^) for 30 min. The groups which were kept out of light were the negative control. Finally, the approach of SEM observation and Live/Dead biofilm were the same as above.

**The Generations of Extracellular and** **Intracellular ROS**

1,3-diphenylisobenzofuran probe was used to analyze the levels of extracellular ROS. In brief, nanofibrous membranes (PLA-HA, PLA) were dispersed in 0.5 mg/mL DPBF solution in the dark, then the mixtures were irradiated under a 470 nm laser (100 mW/cm^2^) for various time periods (0 min, 5 min, 10 min, 15 min, 20 min and 30 min). The generation of ROS was demonstrated by the characteristic absorption (410 nm) decrease of the DPBF using UV-Vis absorption spectrum.

2,7-dichlorofluorescein diacetate (DCFH-DA) was used to detect the ROS levels in *C. auris*. DCFH-DA itself has no fluorescence, intracellular esterase can hydrolyze the intracellular DCFH-DA into the membrane-impermeable agent DCFH, which can be rapidly oxidized by ROS to its fluorescent derivative-dichlorofluorescein (DCF). The ROS levels can be reflected by the fluorescence intensities of DCF [37]. The overnight *C. auris* cultures were diluted to OD_600_ of 0.1 in YPD medium. After incubation to log phase, *C. auris* were treated with nanofibrous membranes (PLA-HA, PLA) for 30 min with or without 470 nm laser (100 mW/cm^2^) irradiation. Then the *C. auris* were incubated in the dark for the subsequent 3 h. Next, *C. auris* cells were collected, washed thrice with PBS, and stained with 10 µM DCFH-DA for 30 min in darkness. After staining, the medium was replaced with PBS. The fluorescence images and green fluorescence intensity of resuspended cells were acquired using fluorescent microscope (BX43, Olympus, Japan) and fluorescent spectrometer (F-7000, Hitachi, Japan).

**Photooxidation of Potassium Iodide**

KI was chosen as a model substrate to investigate the photooxidation activity of PLA-HA membrane. In brief, PLA and PLA-HA membranes were cut into 1 cm × 1 cm. Individual membranes were immersed into 20 mL beakers containing 0.1 M KI in the dark, respectively. Then the mixtures were irradiated under a 470 nm laser (100 mW/cm^2^) for various time periods (0 min, 5 min, 10 min, 15 min, 20 min and 30 min). The absorption of the solution at 287 nm and 352 nm was recorded by a UV-visible absorption spectrometer.

**Apoptotic Detection**

The AnnexinV-FITC apoptosis detection kit was used to distinguish early/late cellular apoptosis according to the manufacturer’s instructions with minor modification. After 470 nm laser induced PDT treatment of nanofibrous membranes as described above, cells were harvested and washed with PBS three times. In order to obtain protoplasts of *C. auris*, the above cultures (1 × 10^6^ CFU/mL) were treated with 2% snailase for 2 h at 30°C. After removing cell walls, cultures were centrifuged softly, washed with sorbitol (1.2 M) three times, and then resuspended in binding buffer (containing 1.2 M sorbitol). To assess the cellular integrity and the externalization of phosphatidylserine (PS), protoplasts of *C. auris* were stained with Annexin-V-FITC and PI for 15 min in the dark at room temperature. Next, the induction of apoptosis was determined by FACS^®^ Caliber flow cytometer (FACSCalibur; Becton Dickinson, San Jose, CA). A total of 10,000 events were counted at the flow rate. Data analysis was performed using Cell Quest software (Becton Dickinson Immunocytometry Systems). All experiments were performed in triplicate.

**Mitochondrial Membrane Potential Assay**

As the hallmark for cellular early apoptosis, mitochondrial membrane potential (MMP) was measured using 5,5’,6,6’-tetrachloro-1,1’,3,3’-tetraethyl-benzimidazolyl-carbocyanine iodide (JC-1) by detecting a switch from red to green fluorescence. After aPDT as described above, the treated *C. auris* cells were harvested, washed, and stained with 2.5 µg/ml JC-1 for 30 min in the dark. After washed with PBS three times, the cells were analyzed by fluorescence spectrophotometer (F-7000, Hitachi, Japan). The ratio of the fluorescence intensities of aggregates JC-1 to monomer JC-1 was calculated.

**Detection of Nuclear Condensation**

Nuclear condensation was examined using 4’,6-diamidino-2-phenylindole (DAPI) staining. Firstly, the cells in four groups were treated as described above. Secondly, the treated cells were collected and washed with PBS three times. Then, cells were fixed in 70% ethanol for 1 h at -4°C. After that, the cells were washed with PBS three times and stained with 2 µg/ml DAPI for 30 min in the dark. Finally, the cells were washed and observed by fluorescent microscope.

**Determination of DNA Fragmentation**

The terminal deoxynucleotidyl transferase (TdT) dUTP nick end labeling (TUNEL) stain is widely used for detecting cell apoptosis. After aPDT as described above, the treated yeast cells were collected and washed with PBS three times. Then, cells were fixed in 3.6% paraformaldehyde for 30 min, and permeabilized in PBS which containing 0.3% Triton X-100 on ice for 2 min. After that, the cells were washed with PBS three times and stained by one step TUNEL assay kit for 1 h at 37°C. Finally, the apoptotic cells were assessed by fluorescent microscope.

**Metacaspase Activation Assay**

CaspACE FITCVAD-FMK in situ marker (Promega) was used to detect the metacaspase activities according to the manufacturer's protocols. After 470 nm laser triggered aPDT of nanofibrous membranes as described above, the treated *C. auris* cells were collected and washed with PBS three times. Then, the cells were stained with 5 µM CaspACE FITC-VAD-FMK at 30°C for 30 min in darkness. Finally, the cells were washed again and analyzed by fluorescent microscope.

**Quantification of Cytochrome C in Cytoplasm and Mitochondrion**

Cytochrome C levels were examined as described previously with minor modification [45]. After aPDT as described above, the treated *C. auris* was harvested. Then, the protoplasts were prepared by the 2% snailase, according to the above methods. After that, the protoplasts were homogenized in buffer A (50 mM Tris, 2 mM EDTA, 1 mM phenylmethylsulfonyl fluoride, pH = 7.5), and the supernatant was collected. Next, the obtained supernatant was centrifuged at 50,000 rpm for 45 min. The supernatant was used to measure the cytochrome C releasing to cytoplasm. On the other hand, the pellet was suspended in buffer B (50 mM Tris, 2 mM EDTA, pH = 5.0) to obtain pure mitochondria. Finally, treated with 500 mg/mL ascorbic acid for 5 min, the cytochrome C content in the cytoplasmic or mitochondrial samples was quantified at a wavelength of 550 nm using a microplate reader (SpectraMax M2).

**Mouse Model of Skin Wound Infection**

All animal experiments were approved by the Institutional Animal Care and Ethics Committee in West China Hospital of Sichuan University (Approval No. 2021621A). Thirty Sprague Dawley (SD) female rats (180-200 g) were purchased from Chengdu Dashuo Biological Technology Co., Ltd, China. Before surgery, the SD rats were adjustable fed for one week to adapt the environment. The rats were anesthetized by intraperitoneal injection of chloral hydrate (10%, 3 mL/Kg). The dorsal hair of mice was removed using a razor and depilatory cream. Then, a full-thickness excisional skin wound (diameter: 20 mm) was created on the dorsum of each rat under sterile conditions. A suspension of *C. auris* (60 µL, 10^6^ CFUs) was inoculated into each wound. After that, all rats were kept in separate cages and raised at 25°C for 2 days. Then, the mice were placed into four groups: *C. auris* infection + PLA-HA membrane + 470 nm illumination group, *C. auris* infection + PLA membrane + 470 nm illumination group, *C. auris* infection + PLA-HA membrane group, and *C. auris* infection + PLA membrane group (n = 6). The observation of *C. auris* infection on the mice wounds was conducted on day 3 and day 21. To measure the fungal burden, the mouse wounds were surgically excised, weighed, and homogenized in PBS. Then, the suspensions containing tissues and *C. auris* were subjected to serial 10-fold dilutions and then spread onto YPD agar plates containing streptomycin and ampicillin. After 48 h incubation at 30°C, the number of colonies was counting. To evaluate the wound contraction rate, a digital camera (EOS 1300D, Canon, Japan) was applied to capture the images of wound beds during the whole healing process. The wound area of each wound was analyzed using *Image J* software.

**Histopathology Assessment**

Briefly, wound tissue and organs from mice in different groups were fixed in formalin solution and embedded in paraffin. Then, tissues were sectioned into 3 µm thick sections. After that, tissue sections were dewaxed in xylene and gradient dehydration using a graded series of ethanol. Finally, sections were stained with hematoxylin and eosin (H&E) and scanned using Pannoramic 250 (3DHISTECH, Hungary). For the periodic acid-Schif (PAS) staining, another set of paraffin blocks were rehydrated using the same method, but they were stained with 1% periodic acid dye (Scy Tech, USA). For immunohistochemical assay, wound tissue sections were labeled using IL-6 antibodies. Briefly, after dewaxing and rehydration, sections were washed with PBS and incubated with 1% hydrogen peroxide for 15 min to deplete endogenous peroxide. Next, sections were placed in sodium citrate buffer, heated in a microwave at 140°C for 3 min, cooled for 15 min at room temperature. Next, sections were blocked with goat serum at 37°C for 20 min and subsequently incubated with primary antibodies against IL-6 at 4°C overnight. Next, sections were washed with PBS, and horseradish peroxidase (HRP) -conjugated secondary antibody (dilution ratio, 1:100) was added for 1 h at room temperature, the immune reaction was visualized by color development with DAB. Finally, sections were counterstained with hematoxylin and scanned using Pannoramic 250. Under the microscope magnifying 400 times, 5 sections of each sample in 3 random sights were selected, and the Image-pro plus 6.0 (Media Cybernetics, Inc., Rockville, MD, USA) was used to conduct the semi-quantitative analysis of integrated optical density (IOD) and pixel area (AREA), the average optical density (AOD) was the ratio of IOD and AREA.

**Statistical Analysis**

The results were analyzed by SPSS software. Every experiment was independently performed at least three times, and the data were expressed as mean ± SD.

# Supporting Information

# Electrospinning

10 wt% PLA was dissolved in the mixture containing DMF and DCM in a ratio of 1:4, After stirring for 12 h at room temperature, 0.2 wt% HA was evenly added into the above mixture and continually stirred for another 2 h at 25°C. The solution was electrospun into nanofibrous membrane through a manual assembly electrospinning system (provided by R&D Lab of Functional Fibers of Sichuan University) with the following parameters: the solution flow rate was 1 mL/h, the range of applied positive and negative voltages were 15 to 20 kV and 0.5-0.6 kV, respectively, the distance between the needle tip and the target was 10-11 cm. All electrospinning processes were conducted under an ambient condition. The obtained nanofibrous membranes were named as PLA (without HA) and PLA-HA.

# Characterization of Electrospun Nanofiber Membranes

The morphology of electrospun nanofibers was observed by a scanning electron microscope (SEM, Hitachi SU3500, Japan) after coating with gold. The average diameter was determined based on the SEM images of each sample, and at least 100 different fiber segments were randomly selected and their diameters were measured using *image J* software. The surface wettability of the nanofibrous membrane surfaces was characterized through a contact angle tester (HarkeSPCAX1, China) using the sessile drop method. The mechanical properties of PLA-HA and PLA were tested by an electronic single yarn strength tester (YM061, China). The thickness between film specimens were not exceed 100%. Also, the tensile rate, width and fixture distance was set on 100 mm/min, 3 mm and 20 mm, respectively. At last, the average of breaking force (cN) and elongation at break (%) were recorded. To measure the imbibition rate and water vapor sorption, squared specimens (1 cm × 1 cm) of each film were cut and obtain the initial mass (W_0_) of each sample using electronic balance. Briefly, the films were soaked with PBS for 12 h, drained by filter paper. Then, the wet mass (Wt) was weighed. The imbibition rate was calculated using the following equation: Imbibition rate (%) = (W_t_ − W_0_) /W_0_ × 100%. For water vapor transmission rate (WVTR), the bottles with ultra-pure water were sealed by nanofibrous films. The moisture consumption through film specimens (M_0_) were recorded. The area of sample (S) and evaporation time (T) was also measured. In this process, humidity and temperature for each test was set for 35%, 55%, 75%, and 21°C, 37°C. Finally, The WVTR (g·m^−2^·d^−1^) was calculated according to the following equation: WVTR (%) = M_0_/S/T × 100%. All experiments were independently repeated in triplicate.

**S1 Fig.** Characterization of nanofibrous membranes. A. The mechanical properties of PLA and PLA-HA. The relationship between breaking strength and elongation at break of PLA and PLA-HA was detected. B&C. The diameter distributions of PLA-HA and PLA. D&E. WVTR of PLA-HA and PLA with different detected humidity and temperature. F. Water contact angle measurements of PLA and PLA-HA. G. The imbibition rate of PLA and PLA-HA. Data are presented as the mean ± s.d.(TIF)

**S2 Fig.** Live/Dead of *C. auris* using Syto9 and PI staining, observing by CLSM.(TIF)

**S3 Fig.** Blood routine examination of *C. auris* after treatment.(TIF)

S1 Table WVTR of PLA-HA and PLA with different detected humidity and temperature.(DOXC)

# Acknowledgements

We thank Prof. Guanghua Huang, Fudan University, for kind supply of *C. auris* strain BJCA001. The authors would like to thank Senior Experimentalist Chaoliang Zhang, State Key Laboratory of Oral Diseases, West China Hospital of Stomatology, Sichuan University, for conducting SEM observations.

**Fundings**

This work was financially sponsored by the National Natural Science Foundation of China (Nos. 81773343, 51803128, 52073186, 81803150), The authors also thank the support by the Post-Doctor Research Project, West China Hospital, Sichuan University (2020HXBH152), 1.3.5 project for disciplines of excellence (ZYJC18033), and HX-Academician project (HXYS19003) of West China Hospital, Sichuan University. The funders had no role in study design, data collection and analysis, decision to publish, or preparation of the manuscript.

# Conflict of Interest

The authors have declared that no competing interests exist.

**Author Contributions**

Conceptualization: Xinyao Liu, Lin Tan, Yuping Ran, Wei Chen

Data curation: Xinyao Liu, Chuan Guo

Formal analysis: Xinyao Liu, Chuan Guo, Kaiwen Zhuang, Lin Tan

Funding acquisition: Yuping Ran, Lin Tan, Kaiwen Zhuang, Xinyao Liu

Investigation: Xinyao Liu, Chuan Guo, Yalin Dai, Muqiu Zhang

Project administration: Xinyao Liu, Chuan Guo

Supervision: Yuping Ran, Lin Tan

Validation: Muqiu Zhang, Yalin Dai, Xinyao Liu

Visualization: Xinyao Liu, Chuan Guo

Writing–original draft: Xinyao Liu

Writing–review & editing: Yuping Ran, Tan Lin, Wei Chen

# Data Availability Statement

The authors declare that the data supporting the findings of this study are available within the paper and the Supporting Information, or are available from the authors upon request.

# References

1.	Brown GD, Denning DW, Gow NAR, Levitz SM, Netea MG, White TC. Hidden killers: human	fungal infections. Sci Transl Med. 2012;4(165):165rv13-rv13. [http://doi.org/	10.1126/scitranslmed.3004404.](http://doi.org/) PMID: 23253612.

2.	Havlickova B, Czaika VA, Friedrich M. Epidemiological trends in skin mycoses worldwide.	Mycoses. 2008;(51):2-15. [http://doi.org/ 10.1111/j.1439-0507.2008.01606.x.](http://doi.org/) PMID: 18783559.

3.	Satoh K, Makimura K, Hasumi Y, Nishiyama Y, Uchida K, Yamaguchi H. *Candida auris* sp. nov.,	a novel ascomycetous yeast isolated from the external ear canal of an inpatient in a Japanese	hospital. Microbiol Immunol. 2009;53(1):41-4. [http://doi.org/ 10.1111/j.1348-0421.2008.00083.x.](../../../Users/Admin/Desktop/快捷方式)	PMID: 19161556.

4.	Chowdhary A, Prakash A, Sharma C, Kordalewska M, Kumar A, Sarma S, et al. A multicentre	study of antifungal susceptibility patterns among 350 *Candida auris* isolates (2009--17) in India:	role of the *ERG11* and *FKS1* genes in azole and echinocandin resistance. J Antimicrob Chemoth.	2018;73(4):891-9. [http://doi.org/ 10.1093/jac/dkx480.](../../../Users/Admin/Desktop/快捷方式) PMID: 29325167.

5.	Chaabane F, Graf A, Jequier LE, Coste AT. Review on antifungal resistance mechanisms in the	emerging pathogen *Candida auris*. Front Microbiol. 2019;(10):2788. [http://doi.org/	10.3389/fmicb.2019.02788.](../../../Users/Admin/Desktop/快捷方式) PMID: 31849919.

6.	Wu D, Wang W, Ng TW, Huang G, Xia D, Yip HY, et al. Visible-light-driven photocatalytic	bacterial inactivation and the mechanism of zinc oxysulfide under LED light irradiation. J Mater	Chem. 2016;4(3):1052-9. [http://doi.org/ 10.1039/C5TA08044D.](../../../Users/Admin/Desktop/快捷方式)

7.	Moro MG, de Carvalho VF, Godoy-Miranda BA, Kassa CT, Horliana ACRT, Prates RA. Efficacy	of antimicrobial photodynamic therapy (aPDT) for nonsurgical treatment of periodontal disease: a	systematic review. Laser Med Sci. 2021;(36):1-18. [http://doi.org/ 10.1007/s10103-020-03238-1.](../../../Users/Admin/Desktop/快捷方式)	PMID: 33438165.

8.	Huang L, Szewczyk G, Sarna T, Hamblin MR. Potassium iodide potentiates broad-spectrum	antimicrobial photodynamic inactivation using Photofrin. ACS Infect Dis. 2017;3(4):320-8.	[http://doi.org/ 10.1021/acsinfecdis.7b00004.](../../../Users/Admin/Desktop/快捷方式) PMID: 28207234.

9.	Huang L, Ma L, Xuan W, Zhen X, Zheng H, Chen W, et al. Exploration of copper-cysteamine	nanoparticles as a new type of agents for antimicrobial photodynamic inactivation. J Biomed	Nanotechnol. 2019;15(10):2142-8. [http://doi.org/ 10.1166/jbn.2019.2829.](../../../Users/Admin/Desktop/快捷方式) PMID: 31462378.

10.	Torabi S, Joharchi K, Kalhori KAM, Sohrabi M, Fekrazad R. Evaluation of antimicrobial	photodynamic therapy on wounds infected by *Staphylococcus aureus* in animal models.	Photodiagn Photodyn. 2021;33:102092. [http://doi.org/ 10.1016/j.pdpdt.2020.102092.](../../../Users/Admin/Desktop/快捷方式) PMID:	33212266.

11.	Hamblin MR. Antimicrobial photodynamic inactivation: a bright new technique to kill resistant	microbes. Curr Opin Microbiol. 2016;(33):67-73. [http://doi.org/ 10.1016/j.mib.2016.06.008.](../../../Users/Admin/Desktop/快捷方式)	PMID: 27421070.

12.	Mima EG, Vergani CE, Machado AL, Massucato EMS, Colombo AL, Bagnato VS, et al.	Comparison of photodynamic therapy versus conventional antifungal therapy for the treatment of	denture stomatitis: a randomized clinical trial. Clin Microbiol Infec. 2012;18(10):E380-E8.	[http://doi.org/ 10.1111/j.1469-0691.2012.03933.x.](../../../Users/Admin/Desktop/快捷方式) PMID: 22731617.

13.	Paramanantham P, Antony AP, Lal SBS, Sharan A, Syed A, Ahmed M, et al. Antimicrobial	photodynamic inactivation of fungal biofilm using amino functionalized mesoporus silica-rose	bengal nanoconjugate against *Candida albicans*. Sci Afr. 2018;(1):e00007. [http://doi.org/	10.1016/j.sciaf.2018.e00007.](../../../Users/Admin/Desktop/快捷方式)

14.	Huang L, Ma L, Xuan W, Zhen X, Zheng H, Chen W, et al. Exploration of Copper-Cysteamine	Nanoparticles as a New Type of Agents for Antimicrobial Photodynamic Inactivation. J	BiomedNanotechnol. 2019;15(10):2142-8. [http://doi.org/ 10.1166/jbn.2019.2829.](../../../Users/Admin/Desktop/快捷方式) PMID:	31462378.

15.	Zhen X, Chudal L, Pandey NK, Phan J, Ran X, Amador E, et al. A powerful combination of	copper-cysteamine nanoparticles with potassium iodide for bacterial destruction. Mater Sci Eng C.	2020;110. [http://doi.org/ 10.1016/j.msec.2020.110659.](../../../Users/Admin/Desktop/快捷方式) PMID: 32204087.

16.	Zhenjun D, Lown JW. Hypocrellins and their use in photosensitization. Photochem Photobiol.	1990;52(3):609-16. [http://doi.org/ 10.1111/j.1751-1097.1990.tb01807.x.](../../../Users/Admin/Desktop/快捷方式) PMID: 2284353.

17.	Fang L-Z, Qing C, Shao H-J, Yang Y-D, Dong Z-J, Wang F, et al. Hypocrellin D, a cytotoxic	fungal pigment from fruiting bodies of the ascomycete *Shiraia bambusicola*. J Antibiot.	2006;59(6):351-4. [http://doi.org/ 10.1038/ja.2006.49.](../../../Users/Admin/Desktop/快捷方式) PMID: 16915819.

18.	Qi SS, Lin X, Zhang MM, Yan SZ, Yu SQ, Chen SL. Preparation and evaluation of hypocrellin A	loaded poly(lactic-co-glycolic acid) nanoparticles for photodynamic therapy. RSC Adv.	2014;4(75):40085-94. [http://doi.org/ 10.1039/c4ra05796a.](../../../Users/Admin/Desktop/快捷方式)

19.	Korbelik M, Madiyalakan R, Woo T, Haddadi A. Antitumor efficacy of photodynamic therapy	using novel nanoformulations of hypocrellin photosensitizer SL052. Photochem Photobiol.	2012;88(1):188-93. [http://doi.org/ 10.1111/j.1751-1097.2011.01035.x.](../../../Users/Admin/Desktop/快捷方式) PMID: 22070614.

20.	Otieno W, Liu C, Deng H, Li J, Zeng X, Ji Y. Hypocrellin B-mediated photodynamic inactivation	of gram-positive antibiotic-resistant bacteria: An in vitro study. Photobiomod photomed.	2020;38(1):36-42. [http://doi.org/ 10.1089/photob.2019.4656.](../../../Users/Admin/Desktop/快捷方式) PMID: 31634029.

21.	Yang Y, Wang C, Zhuge Y, Zhang J, Xu K, Zhang Q, et al. Photodynamic antifungal activity of	hypocrellin A against *Candida albicans*. Front Microbiol. 2019;(10):1810. [http://doi.org/	10.3389/fmicb.2019.01810.](../../../Users/Admin/Desktop/快捷方式) PMID: 31447816.

22.	Jan A, Liu C, Deng H, Li J, Ma W, Zeng X, et al. In vitro photodynamic inactivation effects of	hypocrellin B on azole-sensitive and resistant *Candida albicans*. Photodiagn Photodyn.	2019;(27):419-27. [http://doi.org/ 10.1016/j.pdpdt.2019.07.014.](../../../Users/Admin/Desktop/快捷方式) PMID: 31325521.

23.	Song S, Sun X, Meng L, Wu Q, Wang K, Deng Y. Antifungal activity of hypocrellin compounds	and their synergistic effects with antimicrobial agents against *Candida albicans*. Microb	Biotechnol. 2021;14(2):430-43. [http://doi.org/ 10.1111/1751-7915.13601.](../../../Users/Admin/Desktop/快捷方式) PMID: 32510867.

24.	Yin X, Zhang J, Xu J, Tian M, Li L, Tan L, et al. Fast-acting and highly rechargeable antibacterial	composite nanofibrous membrane for protective applications. Compos Sci Technol.	2021;202:108574. [https://doi.org/10.1016/j.compscitech.2020.108574.](../../../Users/Admin/Desktop/快捷方式) PMID: 29786478.

25.	Lee EJ, KangHuh B, NaKim S, YeonLee J, GwonParkc C, Mikos AG, et al. Application of	Materials as Medical Devices with Localized Drug Delivery Capabilities for Enhanced Wound	Repair. Prog Mater Sci. 2017;(89):392-410. [http://doi.org/ 10.1016/j.pmatsci.2017.06.003.](../../../Users/Admin/Desktop/快捷方式) PMID:	29129946.

26.	Jiang S, Ma BC, Huang W, Kaltbeitzel A, Kizisavas GOU, Crespy D, et al. Visible light active	[http://doi.org/ 10.1039/C8NH00021B.](../../../Users/Admin/Desktop/快捷方式) PMID: 32254131.

27.	Yu Q, Han Y, Tian T, Zhou Q, Yi Z, Chang J, et al. Chinese sesame stick-inspired nano-fibrous	scaffolds for tumor therapy and skin tissue reconstruction. Biomaterials. 2019;(194):25-35.	[http://doi.org/ 10.1016/j.biomaterials.2018.12.012.](../../../Users/Admin/Desktop/快捷方式) PMID: 30572284.

28.	Severyukhina AN, Petrova NV, Yashchenok AM, Bratashov DN, Smuda K, Mamonova IA, et al.	Light-induced antibacterial activity of electrospun chitosan-based material containing	photosensitizer. Mater Sci Eng C. 2017;(70):311-6. [http://doi.org/ 10.1016/j.msec.2016.09.005.](../../../Users/Admin/Desktop/快捷方式)	PMID: 27770896.

29.	Si Y, Zhang Z, Wu W, Fu Q, Huang K, Nitin N, et al. Daylight-driven rechargeable antibacterial	and antiviral nanofibrous membranes for bioprotective applications. Sci Adv. 2018;4(3):eaar5931.	[http://doi.org/ 10.1126/sciadv.aar5931.](../../../Users/Admin/Desktop/快捷方式) PMID: 29556532.

30.	Liu X, Shen X, Fan L, Gao J, Hou C. High-efficiency biosynthesis of hypocrellin A in *Shiraia* sp.	using gamma-ray mutagenesis. Appl Microbiol Biot. 2016;100(11):4875-83. [http://doi.org/	10.1007/s00253-015-7222-9.](../../../Users/Admin/Desktop/快捷方式) PMID: 26767989.

31.	Tort S, Acart Urk FU, Be Csikci A. Evaluation of three-layered doxycycline-collagen loaded	nanofiber wound dressing. Int J Pharmaceut. 2017;529(1-2):642-53. [http://doi.org/	10.1016/j.ijpharm.2017.07.027.](../../../Users/Admin/Desktop/快捷方式) PMID: 28705624.

32.	Adeli H, Khorasani MT, Parvazinia M. Wound dressing based on electrospun PVA/chitosan/starch	nanofibrous mats: Fabrication, antibacterial and cytocompatibility evaluation and in vitro healing	assay. Int J Biol Macromol. 2019;122:238-54. [http://doi.org/ 10.1016/j.ijbiomac.2018.10.115.](../../../Users/Admin/Desktop/快捷方式)	PMID: 30342125.

33.	Zahouani H, Pailler-Mattei C, Sohm B, Vargiolu R, Cenizo V, Debret R. Characterization of the	mechanical properties of a dermal equivalent compared with human skin *in vivo* by indentation	and static friction tests. Skin Res Technol 2009;15(1):68-76. [http://doi.org/	10.1111/j.1600-0846.2008.00329.x.](../../../Users/Admin/Desktop/快捷方式) PMID: 19152581.

34.	Khorasani MT, Joorabloo A, Moghaddam A, Shamsi H, MansooriMoghadam Z. Incorporation of	ZnO nanoparticles into heparinised polyvinyl alcohol/chitosan hydrogels for wound dressing	application. Int J Biol Macromol. 2018;114:1203-15. [http://doi.org/	10.1016/j.ijbiomac.2018.04.010.](../../../Users/Admin/Desktop/快捷方式) PMID: 29634965.

35.	Chen S, Cui S, Hu J, Zhou Y, Liu Y. Pectinate nanofiber mat with high absorbency and	antibacterial activity: A potential superior wound dressing to alginate and chitosan nanofiber mats.	Carbohyd Polym. 2017;174:591-600. [http://doi.org/ 10.1016/j.carbpol.2017.06.096.](../../../Users/Admin/Desktop/快捷方式) PMID:	28821109.

36.	Wang X, Bing J, Zheng Q, Zhang F, Liu J, Yue H, et al. The first isolate of *Candida auris* in	China: clinical and biological aspects. Emerg Microbes Infec. 2018;7(1):1-9. [http://doi.org/	10.1038/s41426-018-0095-0.](../../../Users/Admin/Desktop/快捷方式) PMID: 29777096.

37.	Hou Z, Deng K, Li C, Deng X, Lian H, Cheng Z, et al. 808 nm Light-triggered and hyaluronic	acid-targeted dual-photosensitizers nanoplatform by fully utilizing Nd^3+^-sensitized upconversion	emission with enhanced anti-tumor efficacy. Biomaterials. 2016;101:32-46. [http://doi.org/	10.1016/j.biomaterials.2016.05.024.](../../../Users/Admin/Desktop/快捷方式) PMID: 27267626.

38.	Jin Y, Zhang T, Samaranayake YH, Fang HHP, Yip HK, Samaranayake LP. The use of new probes	and stains for improved assessment of cell viability and extracellular polymeric substances in	*Candida albicans* biofilms. Mycopathologia. 2005;159(3):353-60. [http://doi.org/	10.1007/s11046-004-6987-7.](../../../Users/Admin/Desktop/快捷方式) PMID: 15883718.

39.	Zhang XC, Zhang ZC, Shu QM, Xu C, Zheng QQ, Guo Z, et al. Copper Clusters: An Effective	Antibacterial for Eradicating Multidrug-Resistant Bacterial Infection In Vitro and In Vivo. Adv	Funct Mater. 2021;31(14). [http://doi.org/ 10.1002/adfm.202008720.](../../../Users/Admin/Desktop/快捷方式)

40.	Dominguez EG, Zarnowski R, Choy HL, Zhao M, Sanchez H, Nett JE, et al. Conserved Role for	Biofilm Matrix Polysaccharides in *Candida auris* Drug Resistance. MSphere. 2019;4(1).	[http://doi.org/ 10.1128/mSphereDirect.00680-18.](../../../Users/Admin/Desktop/快捷方式) PMID: 30602527.

41.	Rajendran R, Sherry L, Deshpande A, Johnson EM, Hanson MF, Williams C, et al. A Prospective	Surveillance Study of Candidaemia: Epidemiology, Risk Factors, Antifungal Treatment and	Outcome in Hospitalized Patients. Front Microbiol. 2016;7. [http://doi.org/	10.3389/fmicb.2016.00915.](../../../Users/Admin/Desktop/快捷方式) PMID: 27379047.

42.	Rustamadji P, Wibowo J, Murtani B, Magdalena C. Retraction: Periodic acid-Schiff and alcian	blue immunohistochemistry to detect mucin in mucinous breast carcinoma. Med J Indones.	2020;29(3):346-. [http://doi.org/ 10.13181/mji.ret.205057](../../../Users/Admin/Desktop/快捷方式).

43.	Madeo F, Fr Ohlich E, Ligr M, Grey M, Sigrist SJ, Wolf DH, et al. Oxygen stress: a regulator of	apoptosis in yeast. J Cell Biol. 1999;145(4):757-67. [http://doi.org/ 10.1083/jcb.145.4.757.](../../../Users/Admin/Desktop/快捷方式)	PMID:	10330404.

44.	Wang T, Xu L, Shen H, Cao X, Wei Q, Ghiladi RA, et al. Photoinactivation of bacteria by	hypocrellin-grafted bacterial cellulose. Cellulose. 2020;27(2):991-1007. [http://doi.org/	10.1007/s10570-019-02852-9.](../../../Users/Admin/Desktop/快捷方式)

45.	Jia C, Zhang J, Yu L, Wang C, Yang Y, Rong X, et al. Antifungal activity of coumarin against	*Candida albicans* is related to apoptosis. Front Cell Infect Mi. 2019;(8):445. [http://doi.org/	10.3389/fcimb.2018.00445.](../../../Users/Admin/Desktop/快捷方式) PMID: 30662877.

46.	Tian H, Qu S, Wang Y, Lu Z, Zhang M, Gan Y, et al. Calcium and oxidative stress mediate	perillaldehyde-induced apoptosis in *Candida albicans*. Appl Microbiol Biot.	2017;101(8):3335-45.	[http://doi.org/ 10.1007/s00253-017-8146-3.](../../../Users/Admin/Desktop/快捷方式) PMID: 28224196.

47.	Madeo F, Carmona-Gutierrez D, Ring J, B Uttner S, Eisenberg T, Kroemer G. Caspase-dependent	and caspase-independent cell death pathways in yeast. Bioph Res Co. 2009;382(2):227-31.	[http://doi.org/ 10.1016/j.bbrc.2009.02.117.](../../../Users/Admin/Desktop/快捷方式) PMID: 19250922.

48.	Phillips AJ, Sudbery I, Ramsdale M. Apoptosis induced by environmental stresses and	amphotericin B in *Candida albicans*. P Natl Acad Sci. 2003;100(24):14327-32. [http://doi.org/	10.1073/pnas.2332326100](../../../Users/Admin/Desktop/一晌贪欢). PMID: 14623979.
