# Supplementary material for: A recyclable and light-triggered nanofibrous membrane against the emerging fungal pathogen Candida auris
Source: PLoS Pathog. 2022 May 25;18(5):e1010534. doi: 10.1371/journal.ppat.1010534 (PMC9173615; doi:10.1371/journal.ppat.1010534)
Supplement: Attachment — Submitted filename: Authors responses final.doc [file ppat.1010534.s008.doc]

**PLOS PATHOGENS	4/13/22**

Dear Editors/Reviewers:

On behalf of all the contributing authors, I would like to express our sincere appreciations of your letter and reviewers’ constructive comments concerning our article entitled “**A recyclable and light-triggered nanofibrous membrane against the emerging fungal pathogen *Candida auris***” (Manuscript No.: PPATHOGENS-D-22-00273). These comments are all valuable and helpful for improving our revision. According to the comments, we have made extensive modifications to our manuscript and supplemented extra data to support the conclusions. Additionally, we feel sorry for our poor writings, however, we do invite a friend of us who is a native English speaker from USA help polish our article. Due to our friend’s help, the article was edited extensively and we hope the revised manuscript could be acceptable for you.

In this revised version, changes to our manuscript were all highlighted within the document by using red colored text. Point-by-point responses to the nice editors and two nice reviewers are listed below this letter.

**Part I - Summary**

**Reviewer #1:** We feel great thanks for your recognition and professional review work on our article. As you are concerned, there are several problems that need to be addressed. According to your nice suggestions, we have made extensive corrections to our previous draft.

**Reviewer #2:** Thanks for your help. The previous studies by Yang et al. (2019) and Jia et al. (2019) gave us many [enlightenment](../../../../E:/%25E8%25BD%25AF%25E4%25BB%25B6%25E5%25AE%2589%25E8%25A3%2585%25E7%259B%25AE%25E5%25BD%2595/Dict/8.10.3.0/resultui/html/index.html#/javascript:;) on the design of our experiment and we have cited those articles in our manuscript. Your comments are all of great importance to our article and all of them have contributed a lot to improve the quality of our article.

**Part II - Major Issues: Key Experiments Required for Acceptance
Q1.** Reviewer #2: The authors said that PLA-HA mediated aPDT could generate singlet oxygen. Then whether PLA-HA-induced *C. auris* apoptosis is due to singlet oxygen, which needs to be investigated.

**Response**: Thanks, this is a very good question. According to your nice suggestions, we have added necessary data to support our results in manuscript. On page 19, lines 353-356, page 20, lines 383-384 in our manuscript, we have described the phenomenon that PLA-HA mediated aPDT could generate singlet oxygen and we have detected singlet oxygen in *C. auris* of the PLA-HA aPDT group (Fig 6, panel C). On page 30, lines 586-588, we have described the experimental method.

**Part III – Minor Issues: Editorial and Data Presentation Modifications**

**Q1.** Reviewer #1: Introduction: The authors reviewed *C. auris*, PS, HA, nanofibrous membrane. However, there is a lack of review on the effect of PLA against fungi, and its advantages and disadvantages as nanomembranes. Related literatures (e.g., Composites Part B: Engineering, 2020, 199, 108238; PLoS Pathogens, 2021,17, e1009470.) should be cited in the introduction considering the advances.

**Response**: Good points! Thanks! We have commented and added the advantages and disadvantages of PLA as a substrate in nanomembranes in our revision on pages 6-7, lines 108-117. Also, we have cited the two literatures you mentioned on page 6, line 111 and page 5, line 70, respectively.

**Q2.** Reviewer #1: Fig 3e, the examination of the toxicity of PLA and PLA-HA using mouse fibroblasts was performed in the absence of light. I suggest the authors clarify the effect of light on toxicity.

**Response**: Thanks for your suggestion! We have added necessary data to supplement our results in manuscript. On page 12, lines 216-221, we have clarified the phototoxicity of PLA-HA towards mouse fibroblast cells. And on page 27, lines 519-520, we have updated the method of this experiment. Additionally, we have replaced the [histogram](../../../../E:/%25E8%25BD%25AF%25E4%25BB%25B6%25E5%25AE%2589%25E8%25A3%2585%25E7%259B%25AE%25E5%25BD%2595/Dict/8.10.3.0/resultui/html/index.html#/javascript:;) ‘Fig 3E’ with our new data.

**Q3.** Reviewer #1: The insets in the SEM images of Figure 4e are not clear.

**Response**: Yes, you are right! According to your nice suggestions, we have rearranged the images in Fig 4E of our manuscript. And we have enlarged the insets images of the original Fig 4E.
**Q4.** Reviewer #1: ‘Fig 4e shows ... Compared with other groups, the biofilm turned sparse and the strains were shrunken after treatment of PLA-HA.’ The authors are suggested to provide SEM images with lower magnification in the supplementary file to support this conclusion.

**Response**: Thank you for your reminding, this comment is of great importance to our article. According to your nice suggestions, we have provided SEM images with lower magnification in Fig 4E, and from the image of Light PLA-HA group, the phenomenon of sparse biofilm and shrunken strains can be clearly observed.

**Q5.** Reviewer #2: Please describe the advantage of PLA-HA compared with HA.

**Response**: Thanks for your nice suggestion. In ‘abstract’ section, we have described the advantage of PLA-HA compared with HA on page 3, lines 40-42 according to your suggestion.

Thank you again for your positive comments and valuable suggestions to improve the quality of our manuscript. We hope the revision of the manuscript is suitable for publication in your prestigious journal. Should there been any other corrections we could make, please feel free to contact us.

Thank you for your consideration.

With best regards,

Yuping Ran, M.D.& Ph.D.

Professor of Dermatology, West China Hospital, Sichuan University,
No. 37, Guo Xue Xiang, Wuhou District, Chengdu, 610041, China

Email:[ranyuping@vip.sina.com](mailto:ranyuping@vip.sina.com)
